# Aluminum Coins in Esophagus: A Diagnostic Challenge

**Published:** 2011-07-30

**Authors:** Yousuf A. Khan

**Affiliations:** Department of Paediatric Surgery, National Institute of Child Health Karachi, Pakistan

**Dear Sir**

Esophageal foreign body is a common emergency in children with a peak incidence between 6 months and 3 years of age. The upper esophageal sphincter, the cricopharyngeus is the most frequent site of impaction of foreign bodies. The commonest esophageal foreign bodies reported in the literature are the coins [1, 2].

Ingestion of a coin may or may not be a witnessed event. In either case, the ingested coins may pass through the esophagus without causing any symptom or present with dysphagia. The classic radiographic finding on an antero-posterior chest radiograph is a radio-opaque disk in the upper esophagus suggestive of a coin [3].

The common coins in circulation in Pakistan are of denominations 1, 2 and 5 rupees. Rupee 1 coin (composed of bronze and aluminum and brownish in color) and Rupee 2 coin (composed of brass and aluminum and yellowish in color) were issued in September 1998 by State Bank. While rupee 5 coin (composed of copper and nickel and white in color) was issued later in December 2002. These coins, by composition, were radio-opaque; thus could be detectable by radiographs. In November 2008, State Bank of Pakistan issued Re 1 and 2 aluminum coins of the same diameter and design but of lighter weight because of the low cost at production. The peculiar characteristics of aluminum make these coins faintly visible on usual radiographs and if the radiograph is over exposed, they are almost undetectable (Fig. 1) [4].

**Figure:**
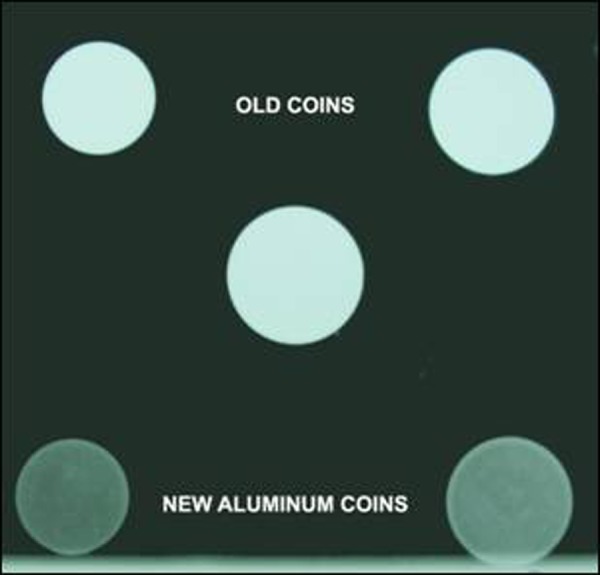
Figure 1: Old and new coins in comparison – radiologically

Two patients presented to us with drooling of saliva, gagging and dysphagia after they ingested coins. The first patient was a 7 years old boy who swallowed a 5 rupee coin 4 hours before presentation. The second patient was a 2 years old girl who swallowed a 2 rupee aluminum coin 6 hours before reporting to ER. Though with a comparable history, there was a remarkable difference in the radiographs of both the patients. X-ray neck and chest revealed a distinct radio-opaque shadow of the coin in the neck area in the first patient, while in second patient the coin was faintly visible (Fig. 2, 3). The coins were removed under direct vision with the help of Magills forceps under general anesthesia (Fig. 4).

**Figure:**
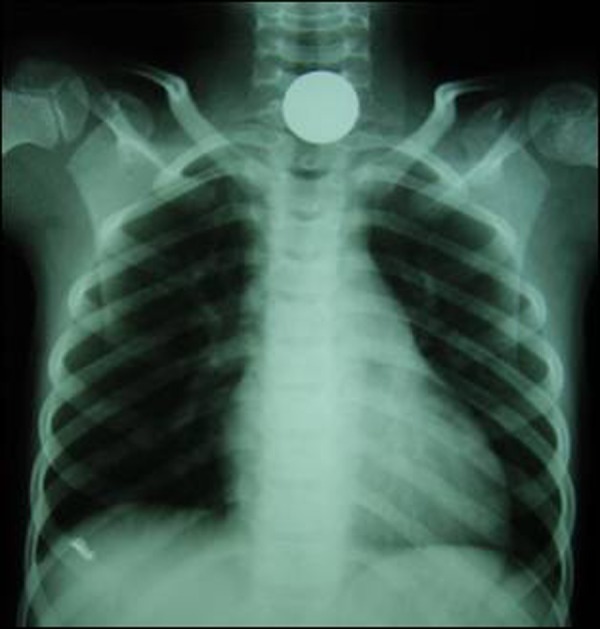
Figure 2: X-ray neck and chest: Showing opaque shadow of a 5 Rupee coin in the cervical esophagus in the 1st patient

**Figure:**
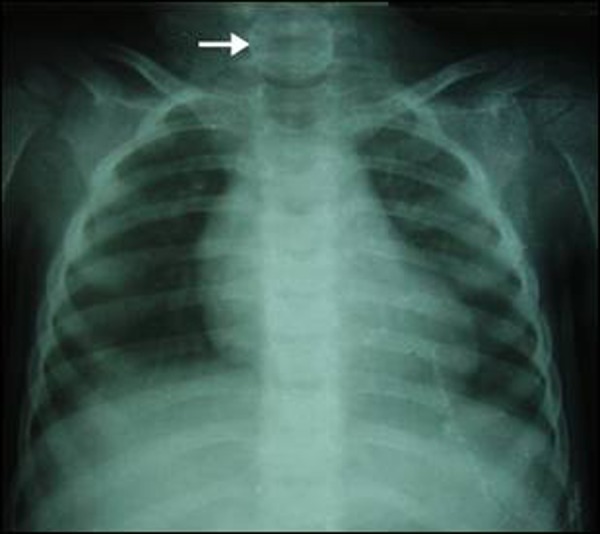
Figure 3: X-ray neck and Chest: showing faintly visible aluminum coin in the cervical esophagus (Arrow) in the 2nd patient

**Figure:**
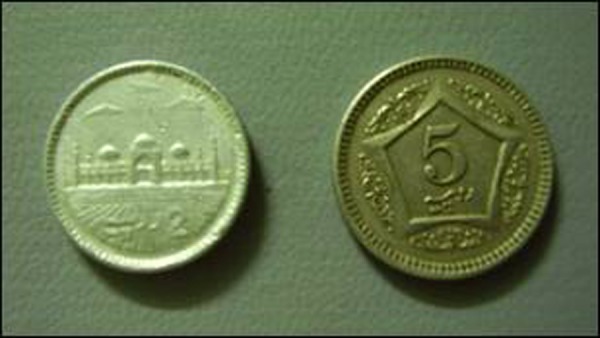
Figure 4: The Coins – retrieved from the esophagus

As these aluminum coins are common in our region, they are a new challenge for all the caregivers. They may be missed easily if one is not vigilant with low quality x-ray and especially when the event is un-witnessed in younger children.

## Footnotes

**Source of Support:** Nil

**Conflict of Interest:** None declared
